# Supplementary material for: Microbial retention and resistances in stormwater quality improvement devices treating road runoff
Source: FEMS Microbes. 2021 Jul 22;2:xtab008. doi: 10.1093/femsmc/xtab008 (PMC10117870; doi:10.1093/femsmc/xtab008)

Supplementary data

Table S1. Insignificance threshold values for groundwater specified by the Länderarbeitsgemeinschaft Wasser (LAWA) in µg/L, which were used to calculate the water pollution index (WPI_{GFS})

Cr	Cu	Ni	Pb	Zn
3.4	5.4	7.0	1.2	60.0

Table S2. List of potential pathogenic taxa identified in road runoff and effluent of stormwater quality improvement devices. ESA: effluent of sedimentation and adsorption, EF: effluent of filtration. All the values are reported as percentages.

Genus	Influent	ESA	EF
Aeromonas	0.07	0.08	0.58
Burkholderia-Caballeronia-Paraburkholderia	0.01	0.03	0.10
Clostridium_sensu_stricto_1	0.08	0.03	0.01
Corynebacterium	0.03	0.16	0.05
Cryptosporangium	-	0.005	-
Enterobacter	0.004	0.001	-
Erysipelothrix	0.015	0.04	-
Enterococcus	-	-	0.006
Escherichia/Shigella	0.001	-	-
Legionella	0.03	0.08	1.91
Mycobacterium	0.04	0.06	0.04
Pseudomonas	2.04	0.47	8.64
Serratia	0.01	0.007	0.009
Staphylococcus	-	-	0.05
Yersinia	0.001	0.003	0.02

Table S3. Results from PERMANOVA analysis to test the effects of environmental parameters on microbial community composition treated runoff by the two SQIDs (effluents, n=20). p-value significance codes: < 0.001 '***'; < 0.01 '**'; < 0.05 '*', 0.05 ".".

		PERMANOVA	
Parameter		**R^2**	**p-value**
pH	20	0.09	0.01**
Intl1	20	0.08	0.03*
Cu	20	0.07	0.05.
Ni	20	0.11	0.0009***
Zn	20	0.09	0.003**

Table S4. Resistance genes encountered on at least six of the sequenced integrons

**Gene**	**# integron sequences**	**Function**	**Database**
czrA	24	Zn and Cd resistance regulator	BacMet
qacE	20	Quaternary ammonium compound-resistance (exporter)	BacMet
czcA	20	Cd, Zn and Co resistance (exporter)	BacMet
mexW	14	Biocide exporter	BacMet
silP	13	Ag efflux system periplasmic chaperone	BacMet
arsB	10	As Sb exporter	BacMet
vexB	9	Multidrug efflux transporter (biocide resistance)	BacMet
merA	7	Hg reductase enzyme	BacMet
acrB	7	Multidrug efflux protein (substrates include biocides as well as the antibiotics ampicillin and chloramphenicol)	BacMet
vexH	6	Multidrug efflux protein (including antibiotics ampicillin and novobiocin)	BacMet
recG	6	DNA repair protein, involved in Cr, Te and Se resistance	BacMet
nia	6	Cation transport ARPase, involved in Fe and Ni resistance	BacMet
adeE	6	Multidrug efflux transporter (biocide resistance)	BacMet
aadA5	6	aminoglycoside nucleotidyltransferase	ResFinder

Figure S1. Structure and treatment processes of the monitored SQIDs. Name of the two SQIDs are shown in green (D1 and D2), main processes are showed in gray boxes, effluents classification are in blue boxes. ESA: Effluent of Sedimentation and Adsorption, EF: Effluent of Filtration. The arrows depict the flow of the water.

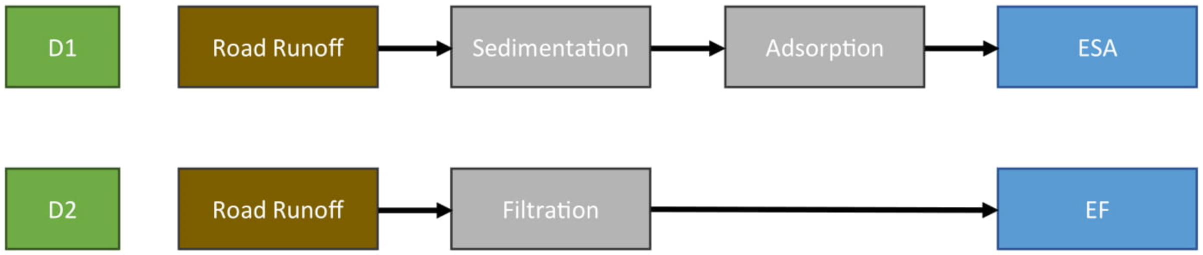

Figure S2. Distribution bar plot of the relative abundance of bacteria community at Phylum (A) and Genus (B) level along different depth layers of filter media samples. Surface (0-5 cm), Medium (5-10 cm), Deep (10-15 cm). For the best representation only taxa with relative abundance > 2% are displayed.

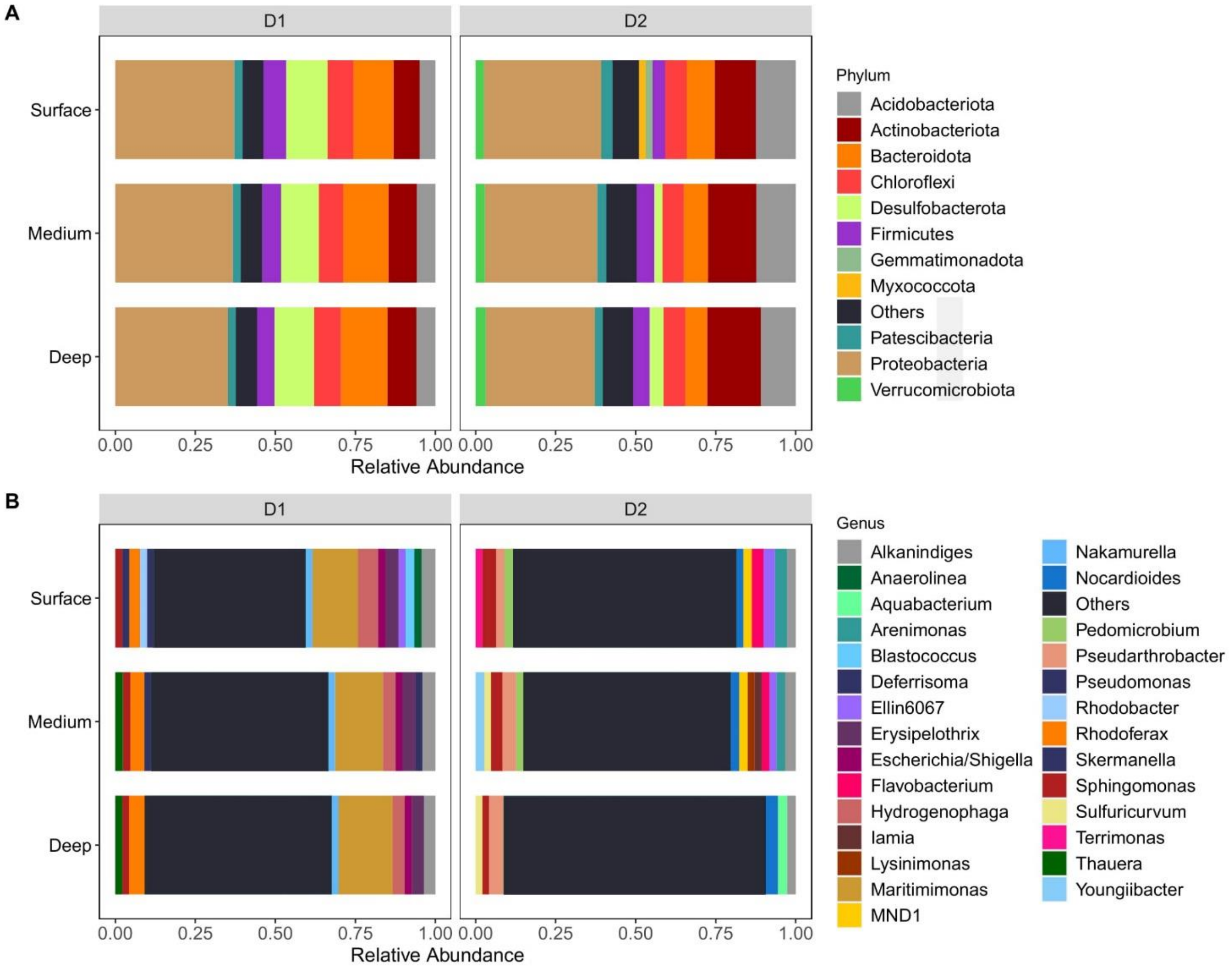

Figure S3. Differential abundance analysis of bacterial relative abundance in D1 (A) and in D2 (B) effluents (n=20) compared to those of the influent. Core taxa with statistically significant difference (log2FC > ± 2; adjusted p-value < 0.05) are displayed in the plot. The different colours depict bacterial phyla. Red arrow indicate taxa present in the influent, black arrow show taxa in the effluents.

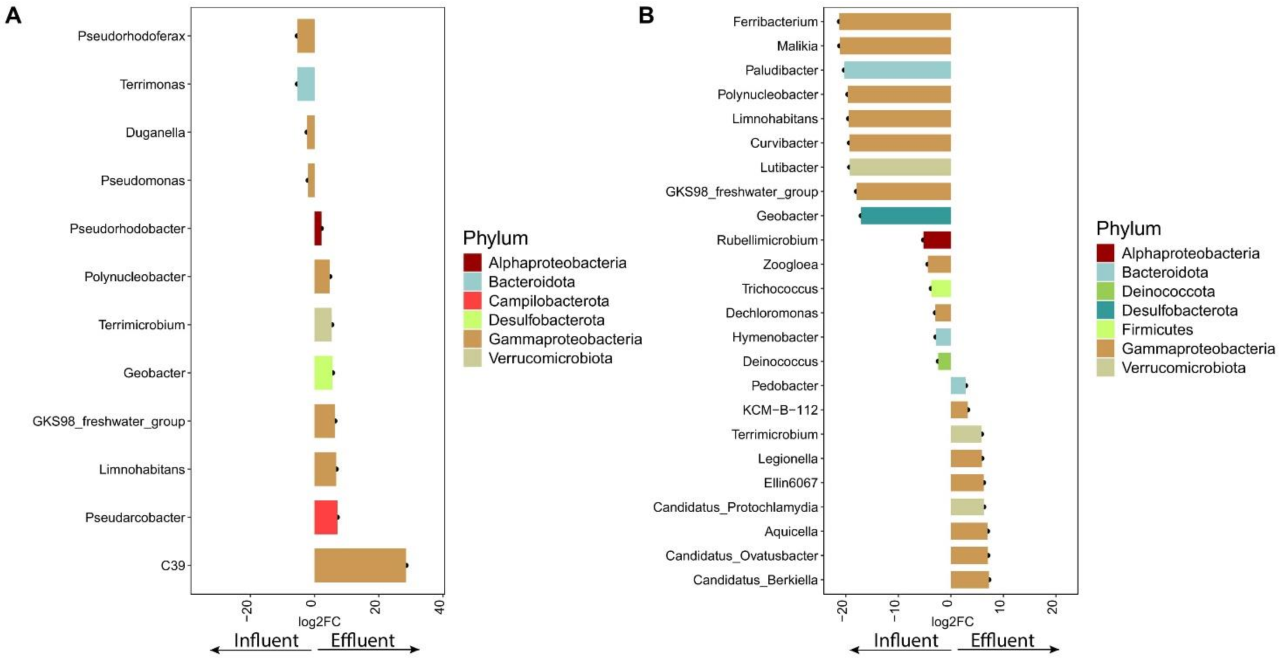

Supplement: xtab008_Supplemental_File [file xtab008_supplemental_file.zip › Liguori_et_al_supplemental_excl_figures_23_03_2021.pdf]
