# Supplementary material for: Microbial retention and resistances in stormwater quality improvement devices treating road runoff
Source: FEMS Microbes. 2021 Jul 22;2:xtab008. doi: 10.1093/femsmc/xtab008 (PMC10117870; doi:10.1093/femsmc/xtab008)
Supplement: xtab008_Supplemental_File [file xtab008_supplemental_file.zip › Transparent Peer Review Information.docx]

**Reviews and Responses**

**Reviewer 1: Anonymous**

The work by Liquori et al. studied the microbial ecology of the stormwater treatment device. The study provided detailed insights into bacterial community structure, antibiotic resistance genes, and relationships with heavy metals as a driving force. Next-generation sequencing techniques (MiSeq and MinIon), together with a qPCR assay were used. This manuscript presented a detailed description of methods used, including necessary quality controls, and limitations were well addressed.

Minor comments are

Line 182, please change the citation format to number

Lines 192-193, how many samples shared the taa with negative controls. Please mention in the results.

Line 211, how much difference is between duplicates of the same samples? Please discuss in the results.

Lines 307, 309, 316 and 319, change to Fig. 1A and Fig. 1B.

Table 2. Column headings (copies x ml) and (copies x g) to change to (copies/ml), and to (copies/g)

**Reviewer 2: Dr Pradip Gyawali**

FEMSMC-2021-019 presents microbial and chemical contaminants along with ARGs present in rainwater samples. The data presented and the conclusion drawn from the findings is reasonable. However, the methodology section lacks vital information. Results were also reported in the method section. LOQs for different chemical contaminants were presented and seems varied with sample metrics. I am not sure they were target limit of quantification or sample limit of quantification.

In Line Cd was used.

Use of LOQ value for samples that were below LOQ may generate bias in results. It should be removed from the analysis or used half of LOQ value. In section 2.3 it is unclear whether DNA was extracted from what. If water than how it was processed.

Negative controls (Water) were used throughout the experimental process. However, results were discarded if contamination was observed. How many samples were discarded? And would that influence the overall findings of the study?

**Author Response to Reviews**

Dear Kathleen Scott,

Thank you very much for handling the review process of our manuscript. We have carfully revised the manuscript adressing all points raised by the reviewers.

Detailed Response

Reviewer: 1

Comments to the Author

The work by Liquori et al. studied the microbial ecology of the stormwater treatment device. The study provided detailed insights into bacterial community structure, antibiotic resistance genes, and relationships with heavy metals as a driving force. Next-generation sequencing techniques (MiSeq and MinIon), together with a qPCR assay were used. This manuscript presented a detailed description of methods used, including necessary quality controls, and limitations were well addressed.

>>> Thank you very much for your constructive review and your comments.

Minor comments are:

1. Line 182, please change the citation format to number

>>> Thank you for noticing. We corrected this.

2. Lines 192-193, how many samples shared the tax with negative controls. Please mention in the results.

>>> A total of 5 samples were sharing major taxa with negative controls, therefore they were removed as false positives from the dataset before the downstream analysis. This has been now indicated in the methods and in the results.

3. Line 211, how much difference is between duplicates of the same samples? Please discuss in the results.

>>> These are technical duplicates used for correcting potential pipetting errors, which we usually not report in the results (similar to duplicates for chemical measurments). We have now amended this in the methods. We assessed the internal variation of our method by looking at the correlation (R2) between the two measurements of duplicate 1 with duplicate 2, which gives us a global estimate of the variance (0.92 in this case, corresponding to 8% variance).

4. Lines 307, 309, 316 and 319, change to Fig. 1A and Fig. 1B.

>>> This has been corrected accordingly

5. Table 2. Column headings (copies x ml) and (copies x g) to change to (copies/ml), and to (copies/g)

>>> Column headings have been changed accordingly

Reviewer: 2

Comments to the Author

FEMSMC-2021-019 presents microbial and chemical contaminants along with ARGs present in rainwater samples. The data presented and the conclusion drawn from the findings is reasonable. However, the methodology section lacks vital information. Results were also reported in the method section.

>>> Thank you very much for your constructive review and your comments. We checked our methodology carefully and redid all affected analysis. The missing details were now presented more clearly in the manuscript.

Comments are:

1. LOQs for different chemical contaminants were presented and seems varied with sample metrics. I am not sure they were target limit of quantification or sample limit of quantification.

>>> That is correct. The LOQs differed due to the applied methods with respect to the sample matrices.

2. In Line Cd was used.

>>> Thank you for noticing. This was a typo error In the text. Cd (Cadmium) has been corrected to Cr (Chromium)

3. Use of LOQ value for samples that were below LOQ may generate bias in results. It should be removed from the analysis or used half of LOQ value.

>>> Thanks for this observation. We adapted the data to 0.5 LOQ as suggested and redid all affected analysis. Yet, the change in the LOQ did not affect the statistical analysis compared to the previous outcome.

4. In section 2.3 it is unclear whether DNA was extracted from what. If water than how it was processed.

>>> We have now included more details in the method section: “Water samples (45 mL) collected from the different devices were centrifuged at 5000 rpm for 10 minutes and the cell pellets were stored at -20 °C, while the filter media samples were directly stored at -20°C until DNA extraction. The DNA from both water (i.e. cell pellets) and filter media samples was extracted using the FastDNA Spin Kit for Soil (MP Biomedicals, Solon, USA), following the manufacturers protocol.”

5. Negative controls (Water) were used throughout the experimental process. However, results were discarded if contamination was observed. How many samples were discarded? And would that influence the overall findings of the study?

>>> A total of 5 samples (out of 92) shared major taxa with the negative controls. They were promptly discarded from the dataset before the analysis as false positives resulting in 86 analyzed samples. The samples discarded from the original dataset should not have any impact on our findings. We have now also stated a sentence in the result section on this.
